# What evidence exists for the use of urban forest management in nature-based carbon solutions and bird conservation. A systematic map protocol

**DOI:** 10.1186/s13750-022-00288-6

**Published:** 2022-10-28

**Authors:** Kayleigh Hutt-Taylor, Carly D. Ziter, Barbara Frei

**Affiliations:** 1 Department of Biology, Concordia University, Richard J. Renaud Science Complex, 7141 Sherbrooke, Canada; 2 Science and Technology Branch Environment and Climate Change Canada, 105 rue McGill, 5th floor, Montreal, QC Canada

**Keywords:** Nature-based solutions, Biodiversity, Avian species, Urban forestry, Climate mitigation, Multiple benefit conservation

## Abstract

**Background:**

There is global interest in finding innovative solutions that address current climate and societal challenges in an urban context. Cities are often on the front lines of environmental change, meaning urban greening strategies have high potential to provide benefits across human communities, while protecting global biodiversity. There is growing consensus that nature-based solutions can provide multiple benefits to people and nature while also mitigating the effects of climate change. Urban forest management is well-suited to a nature-based solutions framework due to the wide variety of services trees provide our communities. Effective approaches to urban forest management also have the potential to promote other forms of urban biodiversity, particularly birds and species at risk. However, studies that integrate strategies for both climate and biodiversity conservation are rare. The goal of this systematic map is to gather and describe information on two desired outcomes of urban forest management: (1) conserving avian diversity and species at risk (2) carbon storage and sequestration (i.e., nature-based climate solutions).

**Methods:**

We will identify relevant articles from two separate searches for inclusion in our systematic map that address (1) urban forestry and avian and species at risk conservation and, (2) urban forestry and carbon storage and sequestration. We will search two bibliographic databases, consult 20 relevant organizational websites, and solicit grey literature through an open call for evidence. Eligibility screening will be conducted at two stages: (1) title and abstract and (2) full text. Relevant information from included papers will be extracted and entered in a searchable, coded database. Synthesis of evidence will describe the key characteristics of each study (e.g., geographic locations, interventions, outcomes, species studied) and identify knowledge gaps and clusters of evidence. Our systematic map will guide further research on opportunities for multiple benefits using nature-based solutions, particularly as they relate to urban forest management. Furthermore, our evidence base will support both management and funding decisions to ensure the effective use of resources for maximum benefits across people and ecosystems.

**Supplementary Information:**

The online version contains supplementary material available at 10.1186/s13750-022-00288-6.

## Background

More than half of global populations reside in cities. For example 7 in 10 Canadians currently live in metropolitan areas and record numbers of individuals are settling into the urban peripheries that surround the urban core [1]. The migration of people towards urban regions is consequently threatening available green spaces. Between 2001 and 2019 almost 40% of Canadian urban green areas were converted to grey (e.g., built infrastructure, impervious surfaces), and continue to decrease with urban growth [2]. Such continued expansion of human activities and the urban built environment have contributed to large-scale habitat loss and fragmentation threatening many species [3, 4]. Consequently, global calls to set sustainability standards have emerged so that individual municipalities up to international governing bodies can properly tackle current climate problems, while also meeting future environmental challenges (food production, shifting weather patterns, increasing temperatures etc.) [5, 6]. As urban communities continue to grow, so do opportunities to address global urban issues like biodiversity loss, climate crises and effective approaches to sustainability [7]. Urban sustainability has thus become one of the most notable issues of our time, with impacts that stretch across research disciplines [6]. This has led to global interest in implementing strategies that address both climate and societal challenges in cities where many environmental threats are amplified.

Since urban areas are often situated within biodiversity hotspots, they are also highly relevant areas for biodiversity conservation strategies [8–10]. Canadian cities provide a good example of such policies taking root through programs like ‘Building Back Better’ and Park People’s City Parks Report which highlight equity and community restoration to advance climate action and sustainability [11, 12]. However, priority conservation areas in Canada are disproportionately located in more heavily settled southern landscapes [13] which represent less than 5% of Canadian land and inland waters yet provide habitat for over 60% of Canada’s species at risk. These heavily settled regions generally have little protected areas or remaining natural habitat [13], despite research increasingly showing the value of even very small habitat patches for biodiversity conservation [14]. Targeting existing policy and conservation tools (e.g., “Species at Risk Act” and “Migratory Birds Convention Act”) through protecting and restoring urban green spaces is a promising strategy to contribute to human wellbeing, biodiversity conservation, and climate resilience. City-based approaches thus have high potential to provide these benefits to human communities while also addressing climate solutions and conservation issues [15].

Urban forest management has often been used as an effective city-based tool for climate change mitigation. Many urban forest management strategies also fall under the umbrella of nature-based solutions (NbS) - defined as strategies that conserve, sustainably manage, and restore ecosystems to benefit both people and biodiversity [16, 17]. Urban trees act as a natural climate solution by fixing carbon and storing it as biomass functioning as a carbon sink [18]. Urban tree planting, in particular, has been identified as a nature-based *climate* solution (NbCS), those NbS specifically targeting climate change resilience through carbon storage and climate change mitigation [17, 19]. For example, long term CO_2_-source and sink dynamics shift as the urban forest changes through time while trees grow, die and decay, however, effectively increasing the number of trees within the urban forest has potential to slow the accumulation of atmospheric carbon [18, 20]. Well informed urban tree planting initiatives can also provide multiple benefits to human communities [21], including those related to climate adaptation and improved health and wellbeing [22]. For example, a recent review of urban tree planting outcomes highlighted tree planting as a most promising climate adaptation [22]. However, poorly planned, large-scale tree planting efforts may also serve to worsen the conditions they are intended to ameliorate [19]. While the role of urban trees in climate change adaptation is increasingly studied, considering the potential of urban forest management to contribute to carbon sequestration and storage is not as well represented in the literature compared to natural forest systems [23] - despite integration into policy and decision-making. For example, policies like Canada’s National Climate Solutions Fund highlight urban tree planting as a crucial nature-based climate solution, however, effective mobilization strategies for such policies will require an integrated approach.

Effective approaches to urban forest management (conservation, planting, removal, etc.) also have the potential to promote other forms of urban diversity, particularly birds and species at risk [24, 25]. Urban green spaces act as crucial stopover habitat for bird species and may be disproportionately important during certain times of year [26]. Moreover, recent work has shown that many migratory birds are attracted to artificial light in flight, meaning urban areas are experiencing increased stopover densities compared to more rural zones [27]. Combined with longer stopover periods during moult migration in the fall, some bird species spend months’ time at temperate stopovers that are equivalent to time spent breeding [28]. Tree cover, presence of native vegetation, green space area and access to water are all particularly important indicators of urban bird diversity [29–31]. For example, large green spaces like woodlots, golf courses, cemeteries and parks represent key bird diversity hotspots within cities [32].

Given that cities can support high numbers of native bird species, management efforts that focus on avian success are both highly relevant and tangible for urban managers [33]. Like many other species, avian species abundances are declining globally and if urban green spaces aren’t managed effectively, these species will face a variety of mitigable risks [34]. Since variation in individual cities’ green space management approaches lead to differences in key factors such as composition and connectivity, there is still high potential for effective intervention at local scales which can scale-up across cities. Furthermore, birds are well studied [25], indicators of environmental health and change [35] and have the highest degree of actionable conservation policies for management outcomes (e.g., Migratory Bird Convention Act), placing them as a strong indicator of biodiversity more broadly in the nature-based solutions framework.

Urban forest management is well-suited to a NbS framework due to the wide variety of services trees provide our communities, in addition to the various forms of diversity trees support. Urban tree planting programs, in particular, have the potential to support avian species and species at risk, in addition to providing climate mitigation often focused on in policies. However, research studies rarely integrate knowledgebase for both species’ conservation and climate solutions. Currently, we are seeing an evident shift towards more integrated approaches to urban biodiversity management that engage with nature to promote multiple benefits [6, 36]. This shift can be seen through the introduction of NbCS and efforts to reduce the tradeoffs and promote synergies in climate solutions by working directly with nature to tackle societal challenges like climate change [36]. This represents a timely opportunity to better integrate our understanding of biodiversity conservation and climate mitigation in urban areas. To our knowledge no systematic review in the field has used a two-pronged approach (e.g., reviewing two bodies of literature) to synthesize opportunities for multiple benefits while identifying tradeoffs. Moreover, no reviews have targeted urban forest management approaches for both climate mitigation and conservation strategies to conserve birds and species at risk.

## Stakeholder engagement

Within our research team we have two advisory researchers, each with individual expertise in urban forestry and avian conservation, respectively. Our team, in collaboration with the expertise and knowledge of Environment and Climate Change Canada team members, will engage with stakeholders and relevant scientific experts. We will ask researchers, organizations, and members of the public to contribute grey literature on this subject through an open call for evidence and information. This systematic map will also contribute to a larger public-facing project aiming to engage with members of the community through an interactive website and database covering topics in urban nature-based solutions.

## Objectives

We propose a systematic map methodology to address these research topics before undertaking a more comprehensive and quantitative synthesis. Systematic maps are a form of synthesis that aim to provide an accurate description of the evidence base, however, they do not aim to provide a quantitative or qualitative answer to a particular question, rather an overview of the research [37]. The goal of our work is to perform a systematic map of the two existing bodies of literature to guide effective deployment of tree planting and habitat restoration (e.g., Canada’s National Climate Solutions Fund) to maximize multiple benefits to birds and species at risk and mobilize the results through a public-facing website and stakeholder engagement. By gathering materials and synthesizing the literature on best practices at a national level on the topic of urban forest management and climate mitigation, paired with species conservation (birds and/or species at risk), we will inform research and urban managers alike on the opportunities for multiple benefits and tradeoffs within urban communities.

### Primary questions

1. What evidence exists on the use of urban forest management strategies for carbon solutions (nature-based climate solutions)?
2. What evidence exists on the use of urban forest management strategies to support urban birds and/or species at risk?

### Aims

We aim to synthesize information from two bodies of literature, (1) urban forest management for carbon storage and sequestration, and (2) urban forest management for bird and/or species at risk. In addition to our primary research questions, we will also address the following sub-questions:

1. What are the main themes in urban ecological research that have addressed urban forest management for climate-solutions (e.g., carbon storage and sequestration).
2. What are the main themes in urban ecological research that have addressed urban forest management strategies to support birds and/or species at risk conservation.
3. What are the current trends, research efforts, are there evidence clusters or knowledge gaps with potential for generating new knowledge.
4. Are there opportunities for multiple benefits across climate mitigation and species (bird or at risk) conservation through urban forest management?

We will discuss the implications of findings 1, 2, 3 for future nature-based solutions linked to urban forest management. Our findings will inform planning, and implementation decisions, for Canada’s 2 Billion Trees Commitment and the Nature Smart Climate Solutions program.

### Components of the primary question (PICO)

In accordance with systematic map practices, the key elements of each component of the research question were defined into four categories: (i) population, (ii) intervention; (iii) comparator; and (iv) outcome. See Table 1.

**Table 1:** Explanation and description of population, intervention, comparator, and outcome (PICO) framework

PICO	Definition	Description
Population 1 Population 2	Populations of subject(s) which are relevant to the review question.	1: Bird species and species at risk within urban areas 2: The urban forest, which consists of all trees, woody species in parks, streetscapes, private land, vacant lots, or other urban green spaces.
Intervention	Variables which impact the populations or to which the populations are exposed	Conservation, management, implementation, recommendations, or intervention strategies that promote species (avian, forest, at risk) success in the urban landscape
Comparator	What the intervention is compared to.	The absence of intervention between sites and/or across time or comparison. No studies will be excluded based on the presence or absence of a comparator.
Outcome	Consequences of the intervention. All relevant variables that can be reliably measured or synthesized.	1: Measure of change in biological outcomes at three levels: (1) population (trends and patterns), (2) community (species richness, diversity) and (3) individual (abundance, fecundity, survival/mortality). 2: Measure of change in carbon storage or sequestration. Improved mitigation of climate change impacts due to urban forest management interventions and tree planting decisions.

## Materials and methods

Our proposed systematic map will follow, as closely as possible, the guidelines provided by CEE (2018), and conform to ROSES reporting standards (i.e., detailed forms for ensuring evidence syntheses report their methods to the highest possible standards; see [38]).

### Searching for articles

Our systematic review will be based on literature searches of published and grey literature using two publication databases: Web of Science Core Collections and Scopus and 20 relevant websites and online databases (Table 2). Websites of all specialist organizations (Table 2) will be manually searched by our team for links or references to relevant publications and data, including grey literature. Additionally, reference sections of relevant reviews from the scoping process will be hand-searched to identify relevant titles that may not be found using the search strategy. We will target sources of grey literature through a call for evidence using mailing lists and social media and through relevant networks of colleagues with expertise.

**Table 2:** List of targetted websites to be searched for each respective topic

Urban Forest	Avian and Species Risk
1. Tree Canada 2. Canadian Institute for Climate Choices 3. Nature Canada 4. Forests Ontario 5. Nature Conservancy Canada 6. David Suzuki Foundation 7. World Wildlife Fund Canada 8. Forest Stewardship Council 9. Arbor Day Foundation 10. National Aboriginal Forestry Association	1. Ducks Unlimited 2. Birds Canada 3. National Audubon Society 4. Partners in Flight 5. American Ornithological Society 6. American Bird Conservancy 7. Bird Life International 8. Nature Canada 9. Cornell Lab for Ornithology: Celebrate Urban Birds 10. Smithsonian Institute: Migratory Bird Center

#### Search string

We compiled a list of potentially relevant search terms (in English) using an initial scoping process and consultation with advisory team members. Search terms were broken into two components: population and intervention for each respective topic. Our team then developed a set of search strings that were modified and refined iteratively through initial scoping in Web of Science Core Collection (WoSCC) to evaluate the sensitivity of the search terms (see supplement) according to a list of ten benchmark papers for each respective search (Table 3).

**Table 3:** Proposed search string for the execution of the searches optimized for Web of Science Core Collections

*Component*	Bird and Species at Risk Component	Forest Climate Component
	***Search String***	
Population	(Urban OR Suburban OR City OR Cities OR Neighbourhood OR Neighborhood OR Borough OR Periurban OR Green_Space$ OR Urbanization) AND (Avian$ Or Bird$ OR Species_at_Risk OR eBird OR Avifauna)	(Urban OR Suburban OR City OR Cities OR Neighbourhood OR Neighborhood OR Borough OR Periurban OR Street$) AND (Forest$ OR Tree$ OR Deciduous OR Coniferous OR Conifer$ OR Green_Space$ OR Greenspace OR Park)
Intervention	(Management OR Conservation OR Recommendation$ OR Protection OR Difference$ OR Support$ OR Impact$ OR Habitat)	(Carbon OR Climate_Change OR Nature_Based_Solution$) AND (Management OR Mitigation OR Planting$ OR Best practice$ OR Conservation OR Recommendation$ OR Strateg* OR Solution$ OR application$ OR Planning OR Regeneration OR Community OR USA OR Canada)

### Article screening and study eligibility criteria

#### Screening process

Our team will screen articles in two stages: (1) title and abstract and (2) full text. All documents found through databases and search engines will be screened at title and abstract. Each article found to be potentially relevant on the basis of title and abstract will be included at this stage of assessment with reviewers tending towards inclusion in cases of uncertainty. Based on this initial scoping exercise, two or more reviewers will use a random subset of 100 abstracts to undergo a consistency check. To ensure repeatable and consistent decision-making, the results of each reviewer will be compared, and all discrepancies will be discussed to understand why inclusion or exclusion was determined. As needed, inclusion criteria will be revised. Literature found through calls for evidence or from reference sections of review articles will be screened at the full text stage and a subset of articles screened by at least two reviewers will be used as a training set.

#### Eligibility criteria

We will use the following predefined criteria when assessing the relevance and deciding on inclusion or exclusion of articles at the full-text stage (see supplementary for more detail). A minimum of two reviewers will undergo a subset of 25 articles during the full-text stage to undertake another consistency check. A minimum Kappa score of ⋝0.6 will be required prior to any further screening. If there is doubt among reviewers, the selected articles will be discussed, by the research team to come to a decision. Justification for inclusion or exclusion will be recorded and a list of studies rejected (with reasoning) during the full text stage will be provided in the supplement material. Reviewers will not screen studies (at title and abstract or full text) for which they are an author.

Each paper must pass each of the following criteria to be included (see supplementary for detailed tables) either by directly providing all the required data or by referring to other studies where supplementary information is presented.

##### Eligible populations

1) Articles must include a component of the urban forest, trees within urban green spaces like streets, parks, vacant lots, private yards, commercial spaces etc. We define trees as all woody vegetation with a diameter at breast height (DBH) above 5 cm.

2) Articles must include bird species (both resident and migratory) and/or species at risk recorded within an urban setting.

##### Relevant type of comparator

No intervention either in space or time.

##### Eligible outcomes

1) Changes in carbon storage or sequestration by the urban forest or its components.

2) Any change in avian or species at risk at the following three levels: (1) community (species richness, diversity) and (2) population (abundance, trends, and patterns), (3) individual (fecundity, survival/mortality).

##### Eligible location

The study must be conducted with a temperate region North (23.5°N to 66.5°N) or South (23.5°S to 66.5°S).

##### Eligible types of study

Any primary study (empirical work) of urban forest, avian or species at risk communities which have assessed the effect of management efforts to improve carbon sequestration or species conservation.

**Language**.

Only English-language literature will be included during the screening stage.

#### Study validity assessment

Given the broad two-pronged approach of this systematic review, the validity of individual studies will not be appraised. We will extract metadata on aspects of study setting and design from included studies to provide a basic overview of the robustness and relevance of the evidence. However, future work extracting this metadata for in depth critical appraisal and synthesis studies would be beneficial.

#### Data coding strategy

Coding and data extracting steps will take place following full-text screening. The following categories of descriptive data will be extracted from each text: (1) bibliographic information; (2) geographical location (country and/or city); (3) species, species group if applicable; (4) intervention details (5) outcome details. To ensure that data is being extracted in a consistent and repeatable manner our review team’s data extraction will be randomly sub-setted (~ 5%) and will be included in a consistency check before full extraction continues. Any discrepancies will be discussed among team members and clarified before continuing.

### Study mapping and presentation

We aim to present two main outputs from this review: (1) a narrative synthesis and (2) a searchable, coded database (MS-Excel). The database will serve as a graphical illustration of the frequency and distribution of studies related to various intervention or management strategies. Each cell (e.g., study) of the database will be colour coded according to criteria like, geographic location, study design, scale of analysis etc. We will use descriptive statistics to quantify factors like number of articles, key characteristics of each study (e.g., locations, species, interventions, green space types, public or private land, study design, management methods) of the evidence compiled. We will identify both knowledge gaps, or areas of evidence that are currently under-represented and require further research, and knowledge clusters, which are areas of evidence that are well-represented and hold potential for full systematic reviews. We will synthesize information and compile summaries in tables and figures.

## Electronic supplementary material

Below is the link to the electronic supplementary material.

Supplementary Material 1: Climate_Initial Scoping

Supplementary Material 2: Roses form

## Data Availability

The datasets generated and or analyzed during our study will be available in a public repository in DRYAD under the systematic map publishing title.
